# Maintenance of cancer stemness by miR-196b-5p contributes to chemoresistance of colorectal cancer cells via activating STAT3 signaling pathway

**DOI:** 10.18632/oncotarget.17971

**Published:** 2017-05-18

**Authors:** Dong Ren, Bihua Lin, Xin Zhang, Yao Peng, Ziyu Ye, Yan Ma, Yangfang Liang, Longbin Cao, Xiangyong Li, Ronggang Li, Lixia Sun, Qiongru Liu, Jinhua Wu, Keyuan Zhou, Jincheng Zeng

**Affiliations:** ^1^ Guangdong Provincial Key Laboratory of Medical Molecular Diagnostics, Key Laboratory of Medical Bioactive Molecular Research for Department of Education of Guangdong Province, Guangdong Medical University, Dongguan, Guangdong Province, 523808, China; ^2^ Department of Orthopedic Surgery, The First Affiliated Hospital of Sun Yat-Sen University, Guangzhou, Guangdong Province, 510080, China; ^3^ Department of Pathology, Jiangmen Central Hospital, Affiliated Jiangmen Hospital of Sun Yat-Sen University, Jiangmen, Guangdong Province, 529030, China; ^4^ Department of Gastroenterology, The First Affiliated Hospital of Sun Yat-Sen University, Guangzhou, Guangdong Province, 510080, China; ^5^ Department of Pathology, Dongguan Hospital Affiliated to Medical College of Jinan University, The Fifth People's Hospital of Dongguan, Dongguan, Guangdong Province, 523905, China; ^6^ Department of Clinical Laboratory, Jiangmen Central Hospital, Affiliated Jiangmen Hospital of Sun Yat-Sen University, Jiangmen, Guangdong Province, 529030, China

**Keywords:** miR-196b-5p, cancer stem cell, chemotherapeutic resistance, STAT3 signaling pathway, CRC

## Abstract

Emerging studies indicated that cancer stem cells represent a subpopulation of cells within the tumor that is responsible for chemotherapeutic resistance. However, the underlying mechanism is still not clarified yet. Here we report that miR-196b-5p is dramatically upregulated in CRC tissues and high expression of miR-196b-5p correlates with poor survival in CRC patients. Moreover, recurrent gains (amplification) contribute to the miR-196b-5p overexpression in CRC tissues. Silencing miR-196b-5p suppresses spheroids formation ability, the fraction of SP cells, expression of stem cell factors and the mitochondrial potential, and enhances the apoptosis induced by 5-fluorouracil in CRC cells; while ectopic expression of miR-196b-5p yields an opposite effect. In addition, downregulation of miR-196b-5p resensitizes CRC cells to 5-fluorouracil *in vivo*. Our results further demonstrate that miR-196b-5p promotes stemness and chemoresistance of CRC cells to 5-fluorouracil via targeting negative regulators SOCS1 and SOCS3 of STAT3 signaling pathway, giving rise to activation of STAT3 signaling. Interestingly, miR-196b-5p is highly enriched in the serum exosomes of patients with CRC compared to the healthy control subjects. Thus, our results unravel a novel mechanism of miR-196b-5p implicating in the maintenance of stem cell property and chemotherapeutic resistance in CRC, offering a potential rational registry of anti-miR-196b-5p combining with conventional chemotherapy against CRC.

## INTRODUCTION

CRC (CRC) is one of the most common malignant cancers, as well as the primary causes of cancer-related deaths worldwide [1, 2]. Despite advances in the early diagnosis and tumor-specific systemic treatment, the recurrence of CRC remains a significant challenge for the majority of patients with CRC, which severely affects the survival periods of CRC patients [3]. The primary issues responsible for the failure of chemotherapy in CRC patients are the existence of cancer stem cells (CSCs) that are the minority population of cells characterized by the capabilities of self-renewal, unlimited proliferation and differentiation into the multiple lineages of cancer cells [4, 5]. Furthermore, emerging evidence showed that CSCs are crucial for the induction of chemotherapeutic resistance. For example, solute carrier family 34 (type II sodium/phosphate cotransporter), member 2 (SLC34A2) induced chemoresistance in via SLC34A2-Bmi1-ABCC5 signaling in breast cancer cells [6]; cancer stem cells marker CD133^+^ contributed to resistance to therapy in hepatocellular carcinoma [7], suggesting that these two cellular processes are intimately linked. Therefore, elucidating the mechanisms that maintain CSCs properties may help to develop novel therapies aimed at eradicating the CSC population so as to improve the efficacy of chemotherapy in patients with CRC.

The Janus kinase/signal transducer and activator of transcription (JAK/STAT) pathway was first identified as a mediator of cytokine signaling in a study of interferon signaling and primarily consisted of three main components: a cell surface receptor JAK and two STAT proteins [8]. After binding to various ligands, such as interleukin-6 (IL-6), interferon (IFN), and IL-10, the activated JAKs engage with cytokine receptors and then phosphorylate tyrosine residues on the receptor, which further recruits STATs to the receptors where STATs are phosphorylated by JAKs. The phosphorylated STATs are released from the receptors and form homo- or hetero- dimers and translocate to the nucleus where they modulate transcription of target genes [9]. Physiologically, the activation of JAK/STAT is tightly controlled by negative regulators, including tyrosine phosphatases, protein inhibitors of activated STAT and suppressor of cytokine signaling (SOCS) [10–12]. Numerous studies have revealed JAK/STAT signaling pathway was indispensable in many aspects of tumorigenesis, including proliferation, apoptosis, angiogenesis, and metastasis [13, 14]. Furthermore, several mechanisms have been reported to be implicated in the aberrant activation of JAK/STAT signaling, including increased production of cytokines and cytokine receptors from the tumor microenvironment, and loss or decreased expression of negative regulators, such as SOCS and tyrosine phosphatases. For example, Grivennikov and Karin reported that autocrine IL-6 as an important activator of oncogenic STAT3 was implicated in lung adenocarcinomas [15]; furthermore, SOCS3 has been found to be suppressed by hypermethylation in lung cancer cells, which promoted the progression of lung cancer [16]. The above evidence implied that aberrant activation of JAK/STAT3 signaling pathway contributed to tumorigenesis and progression of cancer. Therefore, better understanding the specific mechanism of activation of JAK/STAT signaling will facilitate to development anti-cancer therapy for the treatment of cancer.

MiRNAs are short, noncoding RNAs and exert their functions to modulate target genes expression post-transcriptionally via binding to the 3′ untranslated region (3′UTR) of downstream target genes, leading to mRNA degradation and/or translational inhibition [17]. In the physical condition, miRNAs play important roles in many biological processes, including cell apoptosis, proliferation and differentiation [17, 18]. Several lines of evidence indicated that dysregulation of miRNAs, regardless of intracellular or extracellular sources, contributed to the tumorigenesis, progression and metastasis in a variety of cancers [19–27]. In this study, we found that miR-196b-5p is upregulated in CRC tissues and upregulation of miR-196b-5p is associated with poor survival in CRC patients. Moreover, recurrent gains contribute to the miR-196b-5p overexpression in CRC tissues. Overexpressing miR-196b-5p promotes, while silencing miR-196b-5p inhibits the cancer stem cell properties and chemotherapeutic resistance via targeting negative regulators SOCS1 and SOCS3 of STAT3 signaling pathway, leading to the activation of STAT3 signaling. Importantly, miR-196b-5p is detected at significantly higher levels in the serum exosomes of CRC patients compared to the healthy control subjects. Taken together, our results indicate that miR-196b-5p can serve as serum biomarkers for CRC and targeting miR-196b-5p in combination with traditional chemotherapy may be a novel therapeutic strategy for the treatment of CRC.

## RESULTS

### miR-196b-5p is upregulated in CRC and correlated with poor prognosis

**Figure 1:**
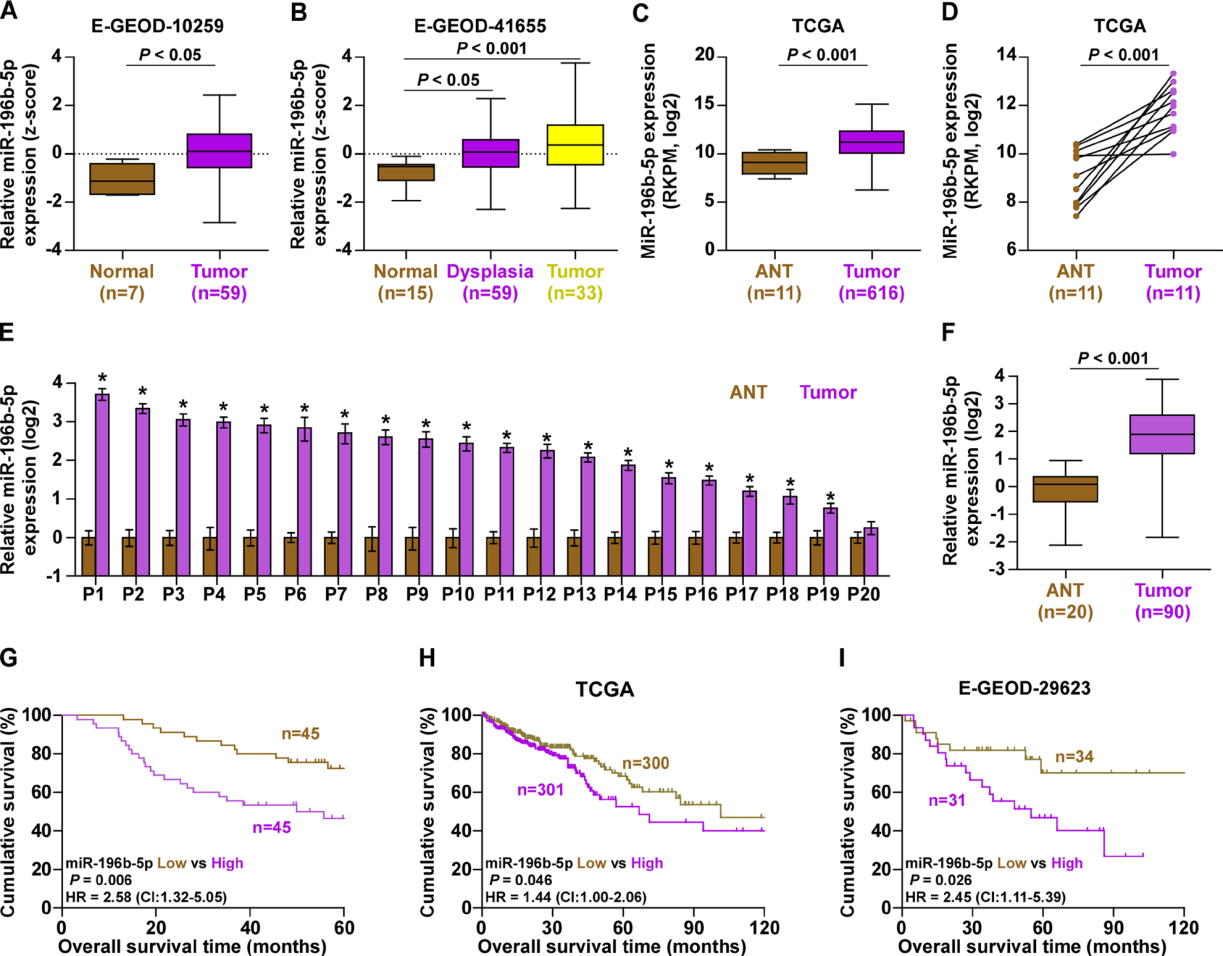
miR-196b-5p is upregulated in CRC and correlated with poor prognosis (**A**–**C**) miR-196b-5p expression levels was markedly upregulated in CRC tissues as assessed by analyzing the E-GEOD-10259, E-GEOD-41655 and TCGA of CRC miRNA sequencing datasets. (**D**) Real-time PCR analysis of miR-196b-5p in 11 primary CRC tissues compared with the matched adjacent normal tissues (ANT). (**E**) Real-time PCR analysis of miR-196b-5p expression in 20 paired collected CRC tissue samples. Transcript levels were normalized to *U6* expression. Each bar represents the mean values ± SD of three independent experiments. *P < 0.05. (**F**) miR-196b-5p expression levels was markedly upregulated in CRC tissues compared with the matched adjacent normal tissues (ANT). (ANT, *n* = 20; CRC, *n* = 90). *P <* 0.001. (**G**) Kaplan–Meier analysis of overall survival curves of patients with CRC with high miR-196b-5p expression (> median, *n* = 45) versus low miR-196b-5p expression (< median, *n* = 45). *P <* 0.001, log-rank test. (**H** and **I**) Kaplan–Meier analysis of overall survival curves of CRC patients datasets from TCGA and E-GEOD-29623.

First, we analyzed high throughput CRC RNA expression profile datasets from ArrayExpress and The Cancer Genome Atlas (TCGA) and found that miR-196b-5p expression was upregulated in CRC tissues compared with normal colorectal tissues and adjacent normal tissues (Figure 1A–1D). It's worth noting that miR-196b-5p was even elevated in the colorectal tissues with dysplasia (Figure 1B), indicating that miR-196b-5p may be implicated in the early neoplastic developmental process of CRC. We further examined miR-196b-5p expression in our own 20 paired CRC tissues. Consistent with TCGA analysis, we found that miR-196b-5p was elevated in CRC tissues and high expression of miR-196b-5p was seen in the 19/20 primary CRC tissue samples compared with the matched adjacent normal tissue samples (Figure 1E). Furthermore, we assessed whether miR-196b-5p expression was clinically correlated with CRC in 90 other CRC tissue samples (Supplementary Table 1: Group1). As shown in Figure 1F, miR-196b-5p expression was markedly elevated in CRC tissue compared with that in 20 adjacent normal colorectal tissues. We further analyzed the correlation of miR-196b-5p expression with clinicopathological characteristics of CRC patients and the result of statistical analysis revealed that increased miR-196b-5p expression positively correlated M-category (*P <* 0.05) (Supplementary Table 2). Importantly, CRC patients with high miR-196b-5p expression had shorter overall survivals (*P* = 0.006; hazard ratio = 2.58, 95% CI = 1.32 to 5.05; Figure 1G) compared with CRC patients with low miR-196b-5p expression, which was consistent with the analysis result from the CRC datasets of TCGA and E-GEOD-29623 (Figure 1H and 1I). Thus, these results suggested that miR-196b-5p is robustly elevated in CRC tissues and high expression of miR-196b-5p correlates with poor prognosis in CRC patient.

### miR-196b-5p targets multiple negative regulators of JAK2/STAT3 signaling pathway

**Figure 2:**
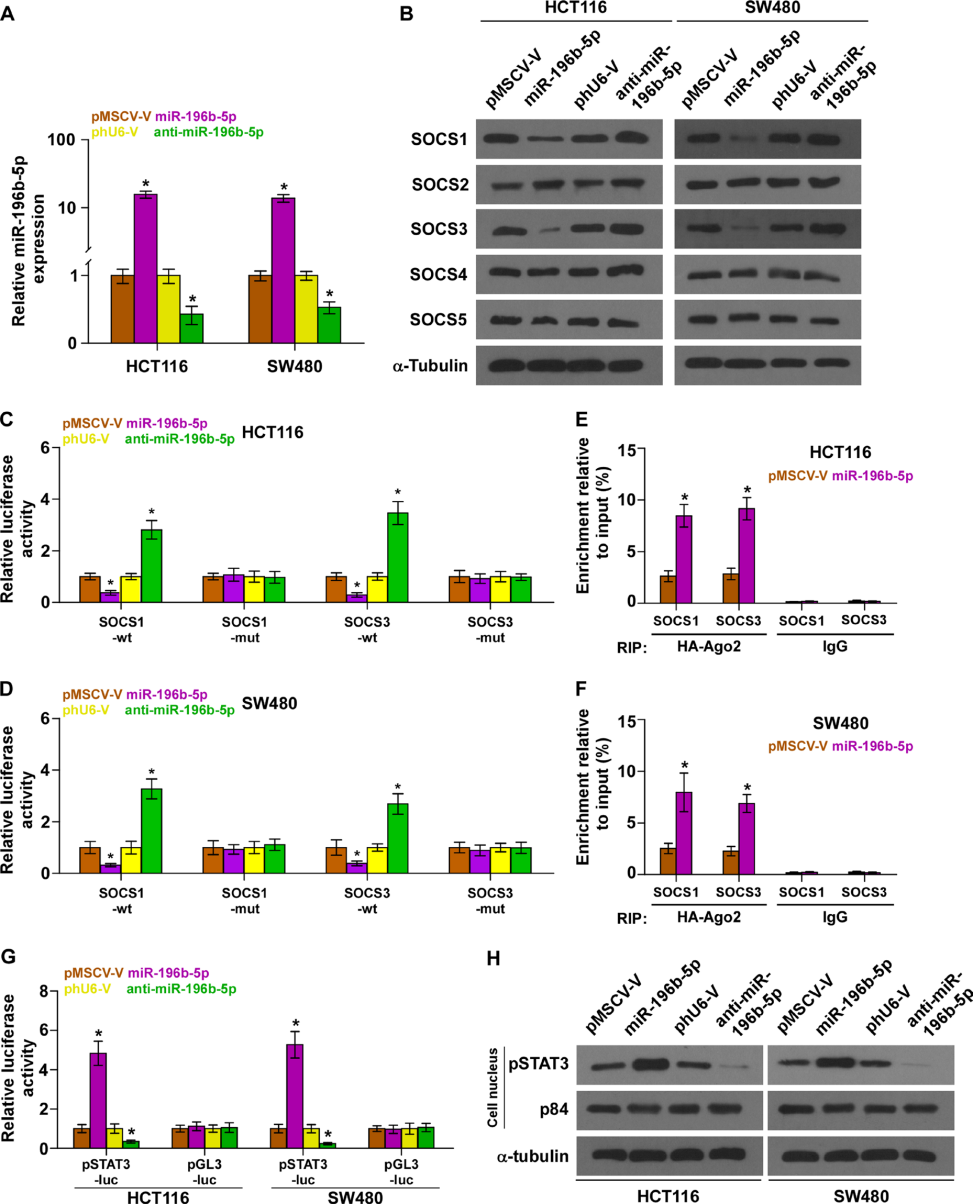
miR-196b-5p activates STAT3 signaling via targeting multiple negative regulators of STAT3 signaling (**A**) Real-time PCR analysis of miR-196b-5p expression in the indicated cells. Transcript levels were normalized by U6 expression. Error bars represent the mean ± SD of three independent experiments. *P < 0.05. (**B**) Western blotting of SOCS1, SOCS2, SOCS3, SOCS4 and SOCS5 expression in the indicated cells. α-Tubulin served as the loading control. (**C** and **D**) Luciferase assay of cells transfected with pmirGLO-3′UTR reporter of SOCS1 and SOCS3 in miR-196b-5p overexpressing and silencing HCT116 and SW480 cells, respectively. Error bars represent the mean ± SD of three independent experiments. *P < 0.05. (**E** and **F**) MiRNP IP assay showing the association between miR-196b-5p and SOCS1,SOCS3 transcripts in HCT116 and SW480 cells. Pulldown of IgGantibody served as the negative control. Error bars represent the mean ± SD of three independent experiments. *P < 0.05. (**G**) STAT3 transcriptional activity was assessed by luciferase reporter constructs in the indicated cells. Error bars represent the mean ± SD of three independent experiments. *P < 0.05. (**H**) Western blotting of nuclear STAT3 expression. The nuclear protein p84 was used as the nuclear protein marker.

Using the publicly available algorithms TargetScan and miRanda, we found that multiple negative regulators of JAK2/STAT3 signaling, including SOCS1, SOCS2, SOCS3, SOCS4 and SOCS5, may be potential targets of miR-196b-5p (Supplementary Figure 1A). We exogenously overexpressed miR-196b-5p via virus transduction, and endogeneously silenced miR-196b-5p by transfecting anti-miR-196b-5p (Figure 2A). Real-time PCR and western blotting analysis revealed that overexpression of miR-196b-5p decreased, while silencing miR-196b-5p increased the mRNA and protein expression levels of SOCS1 and SOCS3, other three members of SOCS families were not affected by miR-196b-5p overexpression or downexpression, indicating that SOCS1 and SOCS3 may be the targets of miR-196b-5p in CRC cells (Supplementary Figure 1B and 1C and Figure 2B). Furthermore, luciferase assay showed that miR-196b-5p overexpression attenuated, while inhibition of miR-196b-5p elevated the reporter activity driven by the 3′UTRs of these transcripts, but not by the mutant 3′UTRs of these transcripts within miR-196b-5p–binding seed regions in HCT116 and SW480 cells (Supplementary Figure 1D and Figure 2C and 2D). Moreover, micro-ribonucleoprotein (miRNP) immunoprecipitation (IP) assay revealed an association of miR-196b-5p with SOCS1 and SOCS3 transcripts (Figure 2E and 2F), further indicating the direct repressive effects of miR-196b-5p on these targets. Collectively, our results suggest that SOCS1 and SOCS3 are authentic targets of miR-196b-5p in CRC cells.

### miR-196b-5p activates STAT3 signaling pathway

We further examined the role of miR-196b-5p in STAT3 signaling pathway in CRC cells. As shown in Figure 2G, miR-196b-5p overexpression in CRC cells significantly increased, while silencing of miR-196b-5p reduced, STAT3-dependent luciferase activity. Furthermore, cellular fractionation and western blotting analysis revealed that overexpression of miR-196b-5p increased nuclear accumulation of STAT3, while silencing miR-196b-5p reduced its nuclear expression, as well as the expression levels of multiple downstream genes of STAT3 signaling pathway including Bcl-2, Bcl-xL and BIRC5 (Figure 2H and Supplementary Figure 2A and 2B). In addition, separate silencing of SOCS1 and SOCS3 rescued the STAT3 activity repressed by miR-196b-5p knockdown, which was more obvious when SOCS1 and SOCS3 were simultaneously silenced (Supplementary Figure 2C), demonstrating that SOCS1 and SOCS3 were functional effectors of miR-196b-5p on regulating STAT3 signaling. Thus, these results demonstrated that miR-196b-5p activates STAT3 signaling pathways via targeting SOCS1 and SOCS3 in CRC cells.

### miR-196b-5p promotes stemness via targeting SOCS1 and SOCS3 in CRC cells

**Figure 3:**
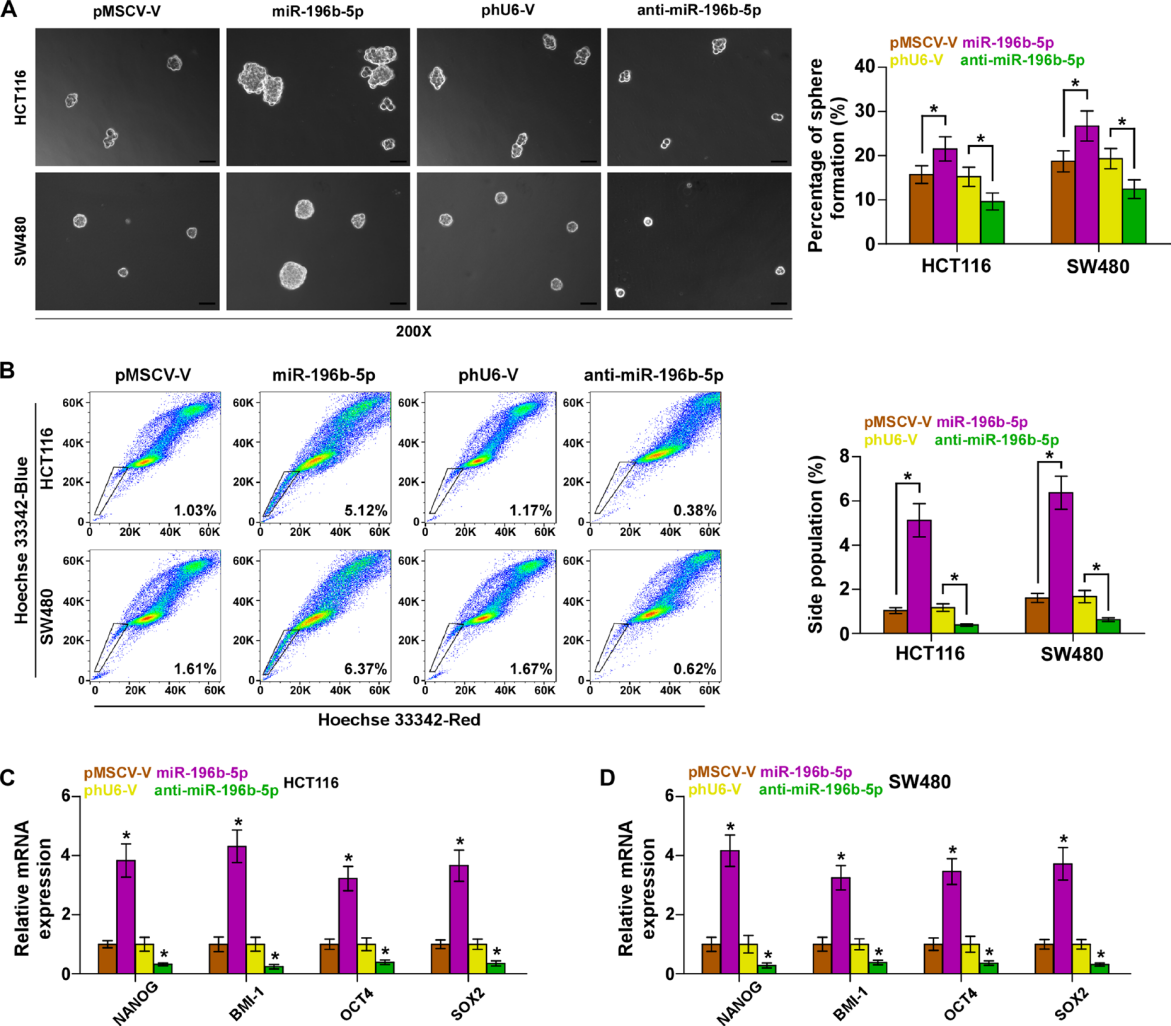
miR-196b-5p promotes stem cell properties in CRC cells (**A**) Representative images of spheroids formed at 200-fold magnification were counted. Histograms showed the mean number of spheroids formed. Scale bars, 50 mm. Error bars represent the mean ± SD of three independent experiments. *P < 0.05. (**B**) Hoechst 33342 dye exclusion assay showed that overexpressing miR-196b-5p promoted the fraction of side population, whereas silencing miR-196b-5p decreased the fraction. Error bars represent the mean ± SD of three independent experiments. *P < 0.05. (**C** and **D**) Real-time PCR analysis of OCT4A, SOX2, NANOG and BMI-1 expression in the indicated cells. GAPDH was used as the loading control. Error bars represent the mean ± SD of three independent experiments. *P < 0.05.

Emerging evidence indicated that STAT3 signaling pathway played important roles in the maintenance of stem cell property and development of chemotherapeutic resistance [13, 14]. We first investigated the effect of miR-196b-5p on spheroids formation and found that overexpression of miR-196b-5p increased spheroids formation ability of CRC cells, while silencing miR-196b-5p decreased spheroids formation ability (Figure 3A). Side population (SP) analysis was carried out and the results revealed that upregulating miR-196b-5p increased, while silencing miR-196b-5p decreased the fraction of SP cells, (Figure 3B). We further measured the expression levels of stem cell factors, including NANOG, BMI-1, OCT4 and SOX2, and found that upregulating miR-196b-5p enhanced, while silencing miR-196b-5p inhibited the expression of these stem cell factors (Figure 3C and 3D). Furthermore, we investigated whether SOCS1 and SOCS3 were implicated in miR-196b-5p-induced stem cell properties. As expected, individual silencing SOCS1 and SOCS3, or simultaneously knocking down SOCS1 and SOCS3 rescued the repressive effects of anti-miR-196b-5p on spheroid formation ablity of CRC cells (Supplementary Figure 2D). Collectively, these results indicated that miR-196b-5p promotes CSC phenotype of CRC cells via targeting SOCS1 and SOCS3.

### miR-196b-5p promotes chemoresistance via targeting SOCS1 and SOCS3 in CRC cells *in vitro*

**Figure 4:**
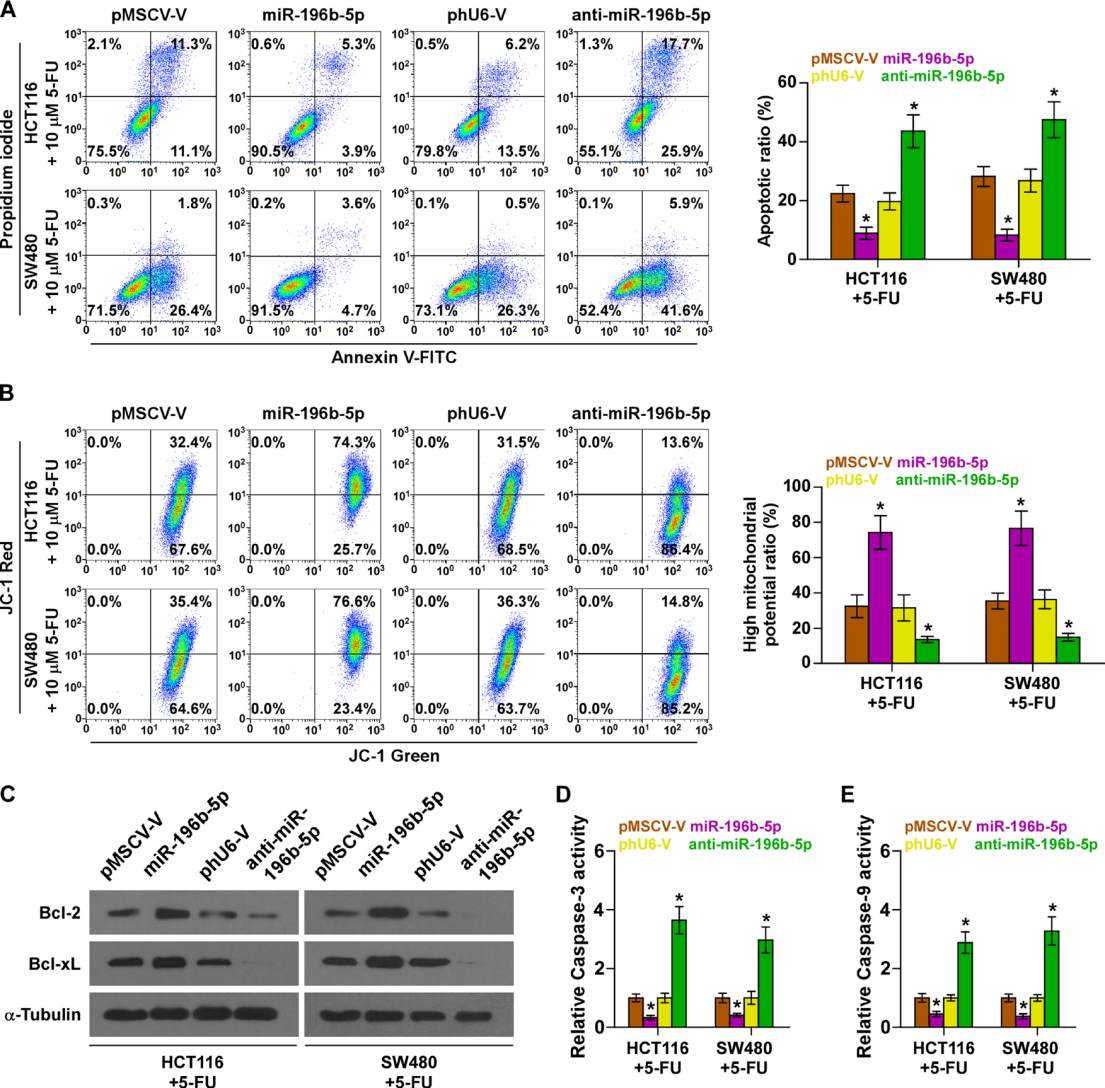
miR-196b-5p promotes chemoresistance in CRC cells *in vitro* (**A**) Annexin V-FITC/PI staining of the indicated cells under treatment of 5-FU. Error bars represent the mean ± SD of three independent experiments. *P < 0.05. (**B**) The JC-1 staining the indicated cells under treatment of 5-FU. Error bars represent the mean ± SD of three independent experiments. *P < 0.05. (**C**) Western blotting analysis of Bcl-2 and Bcl-xL in the indicated cells under treatment of 5-FU. (**D** and **E**) Analysis of the activities of caspase-3 (D) and caspase-9 (E) were detected by the cleaved forms of these two proteins. Error bars represent the mean ± SD of three independent experiments. *P < 0.05.

The effect of miR-196b-5p on the chemoresistance of CRC cells was further investigated. As shown in Supplementary Figure 3A, miR-196b-5p overexpression reduced, while silencing miR-196b-5p increased the apoptosis rate of HCT116 and SW480 cells in the absence of 5-fluorouracil (5-FU) treatment, which was more obvious in treated HCT116 and SW480 cells under treatment of 5-FU (Figure 4A). In addition, miR-196b-5p overexpression increased, while silencing miR-196b-5p decreased, the mitochondrial potential of HCT116 and SW480 cells under treatment of 5-FU (Figure 4B). We further examined the effect of miR-196b-5p on the expression levels of the anti-apoptotic proteins Bcl-2 and Bcl-xL, and the activity of caspase-3 or -9 and found upregulating miR-196b-5p increased Bcl-2 and Bcl-xL expression, but repressed the activity of caspase-3 or -9; conversely, silencing miR-196b-5p decreased Bcl-2 and Bcl-xL expression and increased the activity of caspase-3 or -9 (Figure 4C–4E). The effects of miR-196b-5p on cell growth were not obvious as assessed by MTT assay (Supplementary Figure 3B). Furthermore, individual silencing SOCS1 and SOCS3, or simultaneously knockdown SOCS1 and SOCS3 rescued the repressive effects of anti-miR-196b-5p on 5-FU resistance, as indicated by apoptotic, mitochondrial potential and caspase-3 or -9 activity assays (Supplementary Figure 3C–3F). Collectively, these results indicated that miR-196b-5p promotes chemoresistance of CRC cells to 5-FU via targeting SOCS1 and SOCS3 *in vitro*.

### Downregulation of miR-196b-5p sensitizes CRC cells to 5-FU *in vivo*

**Figure 5:**
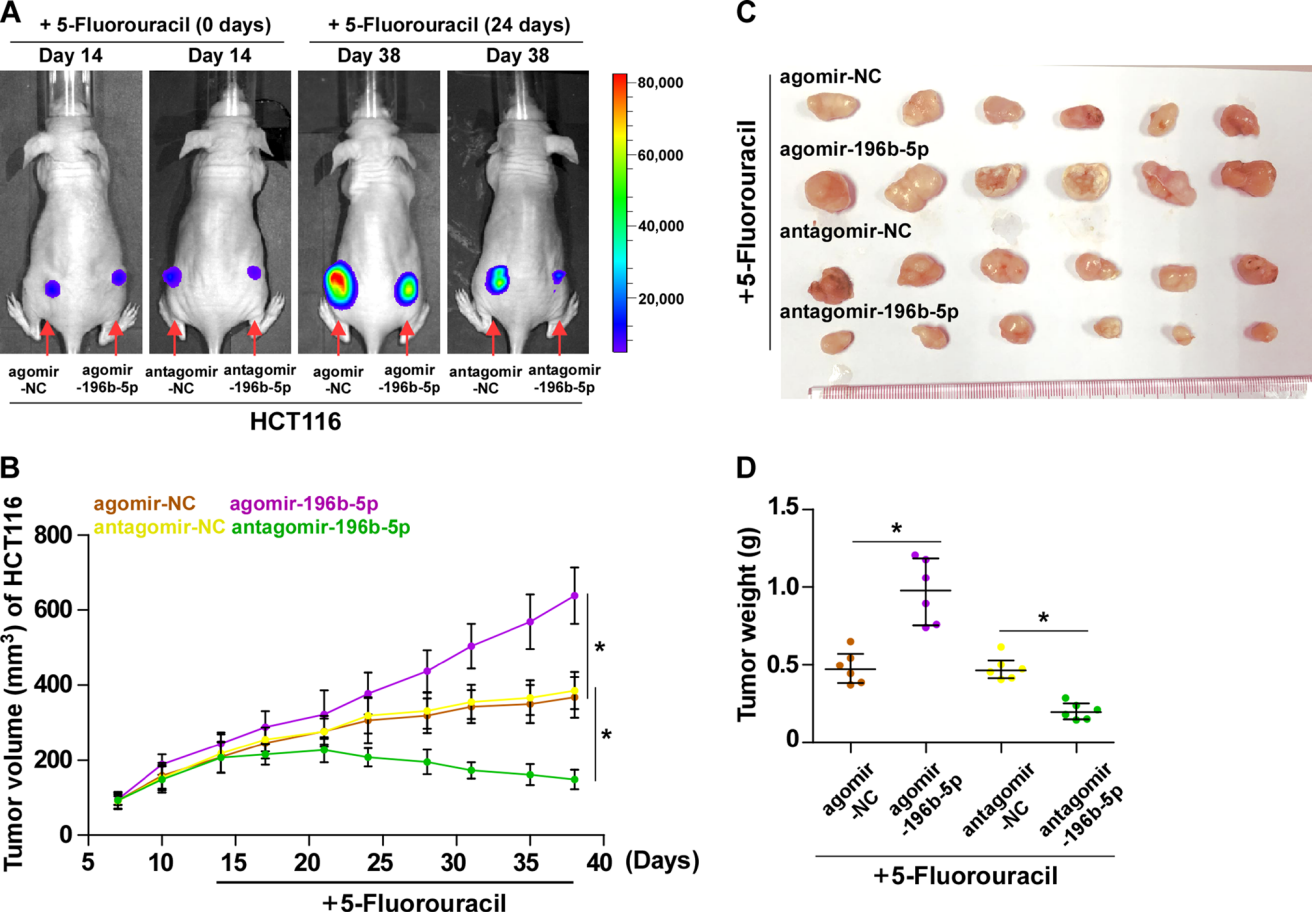
Inhibition of miR-196b-5p sensitizes CRC cells to 5-FU *in vivo* (**A**) Representative images of tumor-bearing mice. The representative images were captured on day 14 and day 38 after inoculating HCT116 cells respectively. The intraperitoneal injection of 5-FU (50 mg/kg.d) started from two weeks after inoculating HCT116 cells and lasted for 24 days, namely 38 day after inoculating HCT116 cells. (**B**) Tumor volumes in the miR-196b-5p–overexpressing, miR-196b-5p–silenced, and control groups were measured on the indicated days. Data presented are the mean ± SD (**C**) Mice were euthanized, and tumors from each experimental group were excised. (**D**) Tumor weights of each group.

We further examined the effect of miR-196b-5p on the chemoresistance of CRC cell *in vivo*. Mice were randomly divided into four groups (*n* = 6/group) and inoculated subcutaneously (3 × 10^6^ HCT116 cells per mouse) in the left dorsal flank. Two weeks later, each group of mice were intratumorally injected with 150 μg agomir negative control, agomir-196b-5p, antagomir negative control and antagomir-196b-5p (2 mg/ml) three times each week for four weeks, combined with intraperitoneal injection of 5-FU (50 mg/kg.d).(Figure 5A). The tumor volumes and weight were increased in the miR-196b-5p-overexpressing plus 5-FU group, but were dramatically decreased in the anti-miR-196b-5p plus 5-FU group, compared to the respective controls (Figure 5A–5D). Collectively, these findings suggest that silencing miR-196b-5p sensitizes CRC cells to 5-FU *in vivo*.

### STAT3 signaling is essential for pro-tumor roles of miR-196b-5p in CRC cells

We then investigated the functional significance of STAT3 signaling in the maintenance of stemness and chemoresistance of CRC cells using STAT3 signaling inhibitors Stattic and S3I-201. As shown in Supplementary Figure 4A, Stattic and S3I-201 dramatically repressed the STAT3 reporter activities in a dose-dependent manner. Notably, the stimulatory effects of miR-196b-5p on STAT3 activity were impaired by these inhibitors (Supplementary Figure 4B). Moreover, inhibition of STAT3 signaling abrogated the spheroid formation ability and protective effect on cell apoptosis by miR-196b-5p overexpression (Supplementary Figure 4C and 4D). These results indicated that activation of STAT3 signaling is critical for miR-196b-5p–induced stemness and chemoresistance in CRC cells.

### miR-196b-5p is dramatically elevated in the serum exosomes in CRC patients

**Figure 6:**
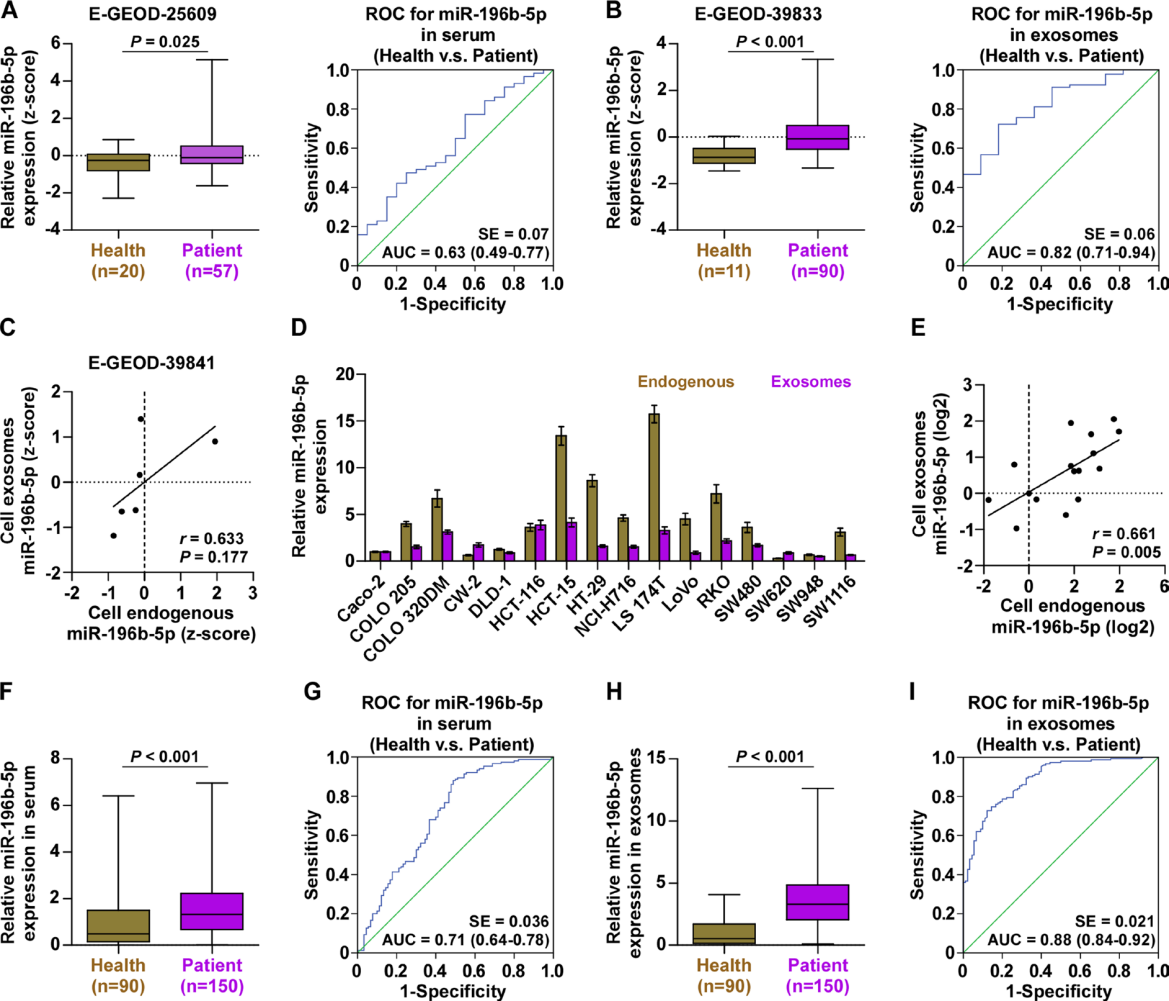
miR-196b-5p is detected in the serum and serum exosomes of CRC patients (**A**) miR-196b-5p expression was elevated in the serum of CRC patients compared with the healthy controls from E-GEOD-25609 dataset (left panel) and ROC curve for miR-196b-5p in the serum of the healthy and CRC patients (right panel). (**B**) miR-196b-5p expression was elevated in the serum exosomes of CRC patients compared with the healthy controls from E-GEOD-39833 dataset (left panel) and ROC curve for miR-196b-5p in the serum exosomes of the healthy and CRC patients (right panel). (**C**) Correlation between mRNA expression levels of miR-196b-5p in 6 different CRC cells and the concentration of miR-196b-5p in their respective exosomes. (**D**) Endogenous expression of miR-196b-5p in diffetent CRC cell lines and miR-196b-5p expression in the exosomes from the supernatant of diffetent CRC cell lines. (**E**) Correlation between mRNA expression levels of miR-196b-5p in different CRC cells and the concentration of miR-196b-5p in their respective exosomes. (**F**) miR-196b-5p expression was elevated in the serum of CRC patients compared with the healthy controls (Health, *n* = 90; CRC, *n* = 150). *P* < 0.001. (**G**) ROC curve for miR-196b-5p in the serum of the healthy and CRC patients. (**H**) miR-196b-5p expression was elevated in the serum exosomes of CRC patients compared with the healthy controls (Health, *n* = 90; CRC, *n* = 150). *P* < 0.001. (**I**) ROC curve for miR-196b-5p in the serum exosomes of the healthy and CRC patients.

As Bollschweiler et al reported that miR-196b-5p was detected as an onco-miR in the exosomes from serum of patients with adenocarcinoma of the esophagus compared with matching primary tumor and normal tissues [28], we analyzed the CRC datasets from E-GEOD-25609 and E-GEOD-39833 and found that miR-196b-5p expression was elevated in the serum and exosomes of CRC patients compared with the healthy controls, and the difference of miR-196b-5p expression in the serum exosomes between cancer patients and the healthy was much more obvious than that seen in the serum (Figure 6A and 6B). Moreover, the expression levels of miR-196b-5p in the exosomes of 6 different CRC cells were consistent with endogenous expression levels of miR-196b-5p in the corresponding cells (Figure 6C). We further examined the miR-196b-5p expression in the exosomes of different CRC cell lines and found that miR-196b-5p expression could be generally detected in the exosomes of all CRC cells (Figure 6D), which was consistent with endogenous expression levels of miR-196b-5p in the corresponding cells (Figure 6E). Moreover, the miR-196b-5p expression levels in the serum and serum exosomes of 150 own CRC patients were examined (Supplementary Table 1: Group2) and 90 healthy controls (Supplementary Table 3), and the results indicated that miR-196b-5p expression in the serum and serum exosomes of CRC patients was significantly elevated compared with healthy subjects, particularly in the exosomes (Figure 6F and 6H). ROC analysis of miR-196b-5p in the serum and serum exosomes of CRC patients presented an AUC of 0.71 (95% CI = 0.64–0.78), Figure 6G) and of 0.88 (95% CI = 0.84–0.92), Figure 6I), respectively. The correlation analysis of miR-196b-5p with clinicopathological characteristics revealed that increased miR-196b-5p expression positively correlated T stage (*P <* 0.05) and M-category (*P <* 0.05) (Supplementary Table 4). Therefore, our results indicated that miR-196b-5p may be identified as a valuable serum biomarker for the diagnosis of CRC.

### Recurrent gains contribute to miR-196b-5p overexpression in CRC tissues

To elaborate the underlying mechanism of miR-196b-5p overexpression in CRC tissues, we further analyzed the CRC dataset from TCGA and found that recurrent gains (amplification) happened in 57.0% of CRC tissues (Supplementary Figure 5A) and expression levels of miR-196b-5p in CRC tissues with gains were dramatically elevated compared with those without gains (Supplementary Figure 5B). We further examined the gains levels in our own 20 paired CRC tissues and 90 individual CRC samples, and found that gains were found in 11/20 paired CRC tissues (55%) and 47/90 CRC tissues (52.2%) (Supplementary Figure 5C and 5D). The expression level of miR-196b-5p in CRC tissues with the gains was robustly higher than those without gains (Supplementary Figure 5E). These results indicate that recurrent gains are responsible for the miR-196b-5p overexpression in CRC tissues.

### Clinical association of miR-196b-5p with SOCS1, SOCS3 and STAT3 signaling activity in human CRC tissues

**Figure 7:**
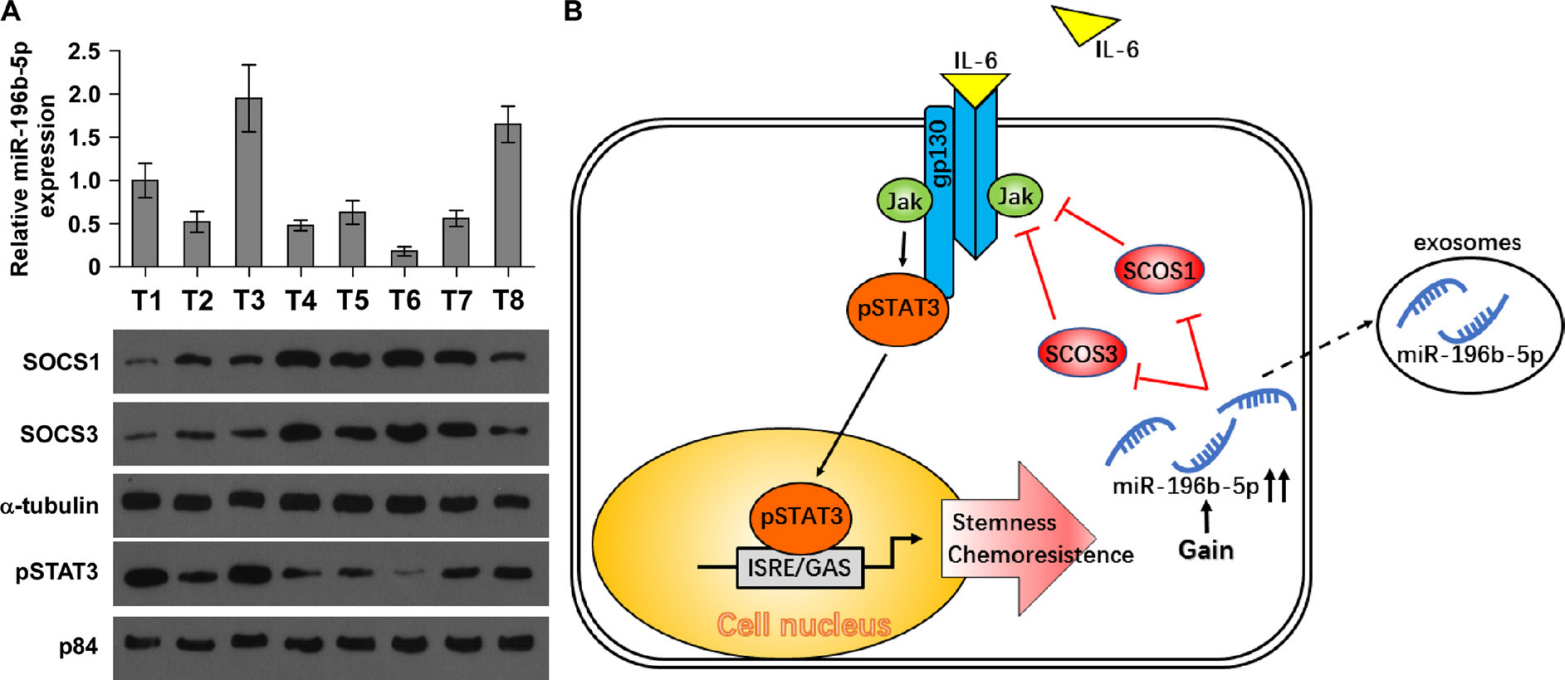
Clinical relevance of miR-196b-5p with SOCS1, SOCS3 and STAT3 signaling activity in human CRC tissues (**A**) Analysis of miR-196b-5p expression with protein expression of SOCS1, SOCS3 and nuclear pSTAT3 in 8 CRC tissues. U6 was used as the control for RNA loading. miR-196b-5p expression levels were normalized to that miR-196b-5p expression of sample one. Loading controls were α-tubulin and p84 for the cytoplasmic and nuclear fractions. Each bar represents the mean ± SD of three independent experiments. *P < 0.05. (**B**) Hypothetical model illustrating that constitutive activation of the STAT3 signaling pathway by miR-196b-5p epigenetic disruption of multiple negative feedback loops contributes to the maintenance of stemness and chemoresistance in CRC cells.

To investigate the clinical correlation of miR-196b-5p with SOCS1, SOCS3 and STAT3 signaling activity, we examined the miR-196b-5p expression and protein levels of SOCS1, SOCS3 and nuclear pSTAT3 in eight frozen human CRC tissues respectively. As shown in Figure 7A and Supplementary Figure 6A–6C, miR-196b-5p expression level in CRC tissues was negatively associated with SOCS1 (*r* = −0.719, *P <* 0.05) and SOCS3 (*r* = −0.707, *P* < 0.05) protein expression, and positively correlated with nuclear pSTAT3 (*r* = 0.745, *P* < 0.05) protein expression. Taken together, these findings demonstrated that overexpression of miR-196b-5p activates STAT3 signaling via directly targeting SOCS1 and SOCS3, which further promotes the stemness and chemoresistance of CRC cells (Figure 7B).

## DISCUSSION

It has been determined that STAT proteins are persistently phosphorylated on the tyrosine residue in the majority of cancers, particularly STAT3, which are required in several aspects of tumorigenesis, including proliferation, apoptosis, increased resistance to chemotherapeutic agents, cancer stem cell and metastasis, leading to the progression of cancer [14, 29, 30]. STAT3 signaling has also been identified to promote the progression and metastasis via inducing cancer stem cell-like properties and chemotherapeutic resistance in different cancers. Marotta and the colleagues reported that the JAK2/STAT3 signaling pathway was required for growth of CD44^+^CD24^−^ stem cell-like breast cancer cells [30]. Furthermore, constitutive activation of STAT3 has been regarded as a prominent mechanism responsible for drug resistance [31]. Notably, some studies have indicated that upregulation of IL-6/JAK/STAT3 signaling is one of the most important pathways involving in colorectal tumorigenesis and plays a pivotal role in the whole developmental processes, including initiation, development and formation in CRC [32]. Inflammatory bowel diseases, including Crohn's disease and ulcerative colitis, are identified to be closely correlated with CRC (called “inflammatory-associated CRC”) where the cytokines, particularly IL-6, drive the development and progression of CRC via downstream activation of JAK/STAT3 signaling pathway, elucidating the oncogenic role of JAK/STAT3 signaling pathway in CRC [33]. However, the molecular mechanisms of JAK/STAT3 signaling activation in CRC are still poorly understood. In this study, we found that miR-196b-5p, which was found to be upregulated in CRC tissues, activated JAK/STAT3 signaling pathway by targeting negative regulators SOCS1 and SOCS3 of JAK/STAT3 pathway. Furthermore, miR-196b-5p promoted cancer stem cell properties and chemoresistance via activating STAT3 signaling pathway *in vivo and in vitro*. Taken together, our results uncover a novel mechanism of miR-196b-5p contributing to the activation of STAT3 signaling in CRC.

A number of studies indicated that several mechanisms have been reported to be implicated in the constitutive activation of JAK/STAT signaling. A few genetic abnormalities have recently been reported in many malignancies, which resulted in the increased STAT tyrosine phosphorylation. In the myeloproliferative disorders, such as polycythemia vera, a somatic activating mutation in the Jak2 kinase resulted in hyperactivation of JAK2 and promoted the development of myeloproliferative disorders [34]. Moreover, increased expressions of cytokines in an autocrine or paracrine manner are involved in the development of tumors. IL-6, the most common and important ligand, was found to be implicated in lung adenocarcinoma in an autocrine manner [15]. Emerging evidence indicated that deregulation of negative regulators of JAK/STAT3 signaling pathway, including tyrosine phosphatase and SOCS families has played crucial roles in the activation of JAK/STAT3 signaling [35, 36]. Of these, increasing attentions have been made about the roles of SOCS families in the regulation of JAK/STAT3 signaling pathway, particularly SOCS1 and SOCS3. SOCS1 and SOCS3 were reported to be involved in negative regulation of the JAK/STAT3 pathway through binding to JAKs and inhibiting their kinase activity [37]. Furthermore, SOCS3 could also inhibit cytokines signal transduction via binding to tyrosine kinase receptors, such as LIF and gp130 [16]. Yoshikawa et al reported that SOCS1 showed growth-suppressive activity and low expression of SOCS1 through methylation of promoter was involved in the development of hepatocellular carcinoma [38]. Moreover, another study indicated that SOCS3 was silenced by hypermethylation in lung cancer, which further promoted the progression of lung cancer [16]. These results indicated that hypermethylation may be the primary mechanism responsible for the decreased expression of SOCS proteins, which contributed to the tumor development and progression via inducing activation of STAT3 signaling. However, the underlying mechanisms that regulate SOCS genes, and the cause of abnormalities in the JAK/STAT signaling pathway in CRC cells, remain largely unknown. In this study, we found members of SOCS families, including SOCS1–5, are the direct targets of miR-196b-5p through bioinformatics. Real-time PCR, western blot, luciferase and RIP assays demonstrated that only SOCS1 and SOCS3 are the *bona fide* targets of miR-196b-5p in CRC cells. Additionally, upregulation of miR-196b-5p enhanced STAT3-dependent luciferase activity in CRC cells, as well as promoted stemness and chemoresistance of CRC cells. Conversely, silencing miR-196b-5p repressed the activity of JAK/STAT3 signaling, which further suppressed stem cell-like phenotypes and reversed the chemoresistance of CRC cells to 5-FU *in vivo and in vitro*, suggesting that targeting miR-196b-5p may be a potential anti-cancer therapeutic strategy against CRC. Furthermore, Xiong and the colleagues reported that hyperacetylation of histones in the SOCS1 and SOCS3 promoters by trichostatin A, a histone deacetylase (HDAC) inhibitor was involved in the upregulation of SOCS1 and SOCS3, which suppressed the growth of CRC cells, and induced the cell apoptosis through downregulating JAK2/STAT3 signaling [39]. Therefore, our results establish a mechanistic link between the inhibition of JAK2/STAT3 signaling and the anti-cancer therapy of targeting miR-196b-5p in CRC cells.

The biological role and clinical significance of miR-196b-5p have been extensively studied and the majority of studies regarding miR-196b-5p converged on the hematologic malignancies, including mixed lineage leukaemia (MLL)-rearranged leukaemia, acute lymphoblastic leukemia, acute myeloid leukemia [40–43]. Furthermore, several studies in solid tumors indicated that miR-196b-5p was up-regulated in many cancers, including glioblastoma, gastric cancer and pancreatic intraepithelial neoplasia [44, 45]. However, another study revealed that ectopic expression of miR-196b-5p abrogated invasion *in vitro* and *in vivo* spontaneous metastasis of breast cancer cells via targeting transcription factor HOXC8, indicating that miR-196b-5p is a potent metastasis suppressors [46]. These findings demonstrated that miR-196b-5p may function as both an oncomir and tumor- suppressive miRNA, depending on the tumor types. However, the biological role and clinical significance of miR-196b-5p in CRC remains largely unknown. In this study, our results found that miR-196b-5p was dramatically elevated in CRC tissues compared to the adjacent normal tissues and high expression of miR-196b-5p correlated with poor prognosis in CRC patients. Consistently, several lines of evidence from the publicly available datasets demonstrated that miR-196b-5p was robustly elevated in CRC tissues in many large cohorts of CRC specimens. Furthermore, upregulating miR-196b-5p promoted, while silencing miR-196b-5p attenuated the stemness and resistance of CRC cells to 5-FU *in vitro and in vivo*, further elucidating the oncogenic role of miR-196b-5p in CRC. Notably, Boisen et al reported that higher expression of miR-196b-5p predicted improved outcome in CRC patients after treated with capecitabine and oxaliplatin with or without Bevacizumab[47]. This may be because rapidly dividing cells are more susceptible to chemotherapy and have better chemotherapy response leading to improved outcomes of cancer patients, suggesting that miR-196b-5p may enhance the proliferation rate of CRC cells. However, the specific mechanism responsible for the improved outcomes in CRC patients with high miR-196b-5p were not mentioned in this manuscript.

Recent literatures indicated that miRNAs regulated the tumor progression and metastasis in an exosome-mediated miRNAs transfer manner, as well as play important roles in modulating the tumor microenvironment [48, 49]. Exosomes are small (∼100 nm) cell-derived membrane-bound extracellular vesicles released by almost all eukaryotic cells into biological fluids, including blood, urine, and cultured medium of cell cultures [50, 51]. However, in cancer patients, circulating exosomes were identified to be much higher than healthy individuals [52, 53], indicating that exosomes may play a functional role in the development of tumors [54]. Indeed, the role of exosomes-mediated miRNA transfer in promoting the tumor recurrence and metastasis by interacting with recipient ells is well-established [26, 55–57]. Furthermore, exosome-delivered miRNAs have also been reported to promote the progression and distant metastasis of CRC. Chiba and the colleagues reported that exosome transfer of miR-21 from SW480 CRC cells promoted the migration ability of HepG2 hepatocellular cancer cells via suppressing their target genes phosphataseand tensin homolog (PTEN) [58]; furthermore, HCC-derived exosomes mediated miRNA transfer has been reported to be an important mechanism of environmental modulation of HCC growth and progression [59]. It's interesting to find that miR-196b-5p was detected in the exosome from serum of patients with esophageal adenocarcinoma [28]. Therefore, we analyzed two publicly accessible CRC datasets and found that miR-196b-5p expression was elevated in the serum and serum exosomes of CRC patients compared with healthy controls, and the difference of miR-196b-5p expression in the serum exosomes between cancer patients and the healthy was much more obvious than that seen in the serum. We further examined the miR-196b-5p expression in the serum and serum exosomes from our own CRC patients. Consistently, we found that miR-196b-5p expression in the serum and serum exosomes of CRC patients was significantly elevated compared with healthy subjects, particularly in the exosomes. However, whether exosome-miR-196b-5p complex just serves as serum biomarker for CRC, or if it does have a role, the specific biological function of exosome-miR-196b-5p complex is still to be further studied.

In summary, our findings reveal that miR-196b-5p plays an important role in the stemness and chemotherapy resistance of CRC cells via activating STAT3 signaling pathway. Importantly, miR-196b-5p was highly enriched in the serum exosomes of patients with CRC compared to healthy control subjects. Therefore, improved understanding of the specific role of miR-196b-5p in the pathogenesis of CRC facilitates to increase our knowledge of CRC development, which will help to develop new therapeutic measures against CRC.

## MATERIALS AND METHODS

### Cell lines and cell culture

The human CRC cell lines Caco-2, COLO 205, COLO 320DM, CW-2, DLD-1, HCT15, HCT116, HT-29, NCI-H716, LS 174T, LoVo, RKO, SW480, SW620, SW948 and SW1116 were obtained from Shanghai Chinese Academy of Sciences cell bank (China) and were cultured in RPMI-1640 medium (Life Technologies, Carlsbad, CA, US) supplemented with penicillin G (100 U/mL), streptomycin (100 mg/mL) and 10%fetal bovine serum (FBS, Life Technologies). All cells were incubated at 37°C in a humidified atmosphere with 5% CO_2_ and were routinely sub-cultured using 0.25% (w/v) trypsin-ethylenediaminetetraaceticacid solution.

### Patients, tumor tissues and serum samples

90 individual CRC tissues and the 20 adjacent tumor normal tissues were obtained during surgery at Clinical Biobank of Collaborative Innovation Center for Medical Molecular Diagnostics of Guangdong Province, Jiangmen Central Hospital (Guangdong, China) between January 2009 and December 2011. 90 serum samples from the healthy and 150 serum samples from CRC patients were obtained at Clinical Biobank of Collaborative Innovation Center for Medical Molecular Diagnostics of Guangdong Province, Jiangmen Central Hospital (Guangdong, China) in 2015. Patients were diagnosed based on clinical and pathological evidence, and the specimens were immediately snap-frozen and stored in liquid nitrogen tanks. For the use of these clinical materials for research purposes, prior patients' consents and approval from the Institutional Research Ethics Committee were obtained. The proportions of tumor vs. non-tumor in H&E-staining tissue samples were evaluated by the two independent professional pathologists. The tumor proportions in all clinical CRC tissue samples analyzed in this study exceeded 70%.

### RNA extraction, reverse transcription, and real-time PCR

Total RNA from tissues or cells was extracted using RNA Isolation Kit-miRNeasy Mini Kit (Qiagen, USA) according to the manufacturer's instructions. Messenger RNA (mRNA) and miRNA were reverse transcribed of total mRNA using the Revert Aid First Strand cDNA Synthesis Kit (Thermo, USA) according to the manufacturer's protocol. Complementary DNA (cDNA) was amplified and quantified on CFX96 system (BIO-RAD, USA) using iQ SYBR Green (BIO-RAD, USA). The primers were provided in Supplementary Table 5. Primers for U6 and miR-196b-5p (Cat#: miRQ0001080) were synthesized and purified by RiboBio (Guangzhou, China) (http://www.sirna.cn/siteen/Products.aspx?id=181). U6 or glyceraldehyde-3-phosphate dehydrogenase (GAPDH) was used as endogenous controls. Relative fold expressions were calculated with the comparative threshold cycle (2^−ddCt^) method.

### Plasmid, small interfering RNA and transfection

The human miR-196b-5p was PCR-amplified from genomic DNA and cloned into a pMSCV-puro retroviral vector (Clontech, Japan). The pSTAT3-luc and control plasmids (Clontech, Japan) were used to examine the activity of transcription factor quantitatively. The 3′ UTR region of the human SOCS1 and SOCS3 were PCR-amplified from genomic DNA and cloned into pmirGLO vectors (Promega, USA), and the list of primers used in clone reactions was presented in Supplementary Table 6. Agomir-196b-5p (Cat#: miR40001080), antagomir-196b-5p (Cat#: miR30001080), small interfering RNA (siRNA) for SOCS3 and SOCS1 and respective control RNA were synthesized and purified by RiboBio. Transfection of miRNA, siRNAs, and plasmids were performed using Lipofectamine 3000 (Life Technologies, USA) according to the manufacturer's instructions.

### Western blotting analysis

Nuclear/cytoplasmic fractionation was separated by using Cell Fractionation Kit (Cell Signaling Technology, USA) according to the manufacturer's instructions, and the whole cell lysates were extracted using RIPA Buffer (Cell Signaling Technology). Western blot was performed according to a standard method, as described previously [60]. Proteins were visualised using ECL reagents (Pierce, USA). Antibodies against Bcl-2 (Cat#: 2872), Bcl-xL (Cat#: 2764), pSTAT3 (Cat#: 9145), SOCS1 (Cat#: 3950), SOCS2 (Cat#: 2779), SOCS3 (Cat#: 2923) were purchased from Cell Signaling Technology and p84 (Cat#: PA5-27816), SOCS4 (Cat#: PA5-21599) and SOCS5 (Cat#: PA5-21600) from Invitrogen. The membranes were stripped and reprobed with an anti–α-tubulin antibody (Cell Signaling Technology. Cat#: 2125) as the loading control.

### Isolation of exosomes

miRNA was extracted from serum using miRNeasy Serum/Plasma Kit (Cat# 217184, Qiagen) according to the manufacturer's protocol. Both isolation of exosomes from serum samples and extraction of miRNA from exosomes were using exoRNeasy Serum/Plasma Maxi Kit (Cat# 77064, Qiagen) according to the manufacturer's protocol. Briefly, precipitated from 400 μL serum and 100 μL exoRNeasy Serum/Plasma Maxi Kit exosome precipitation solutions, exosomes pellets were then dissolved in 200 μL of RNase-free water and examined for further miRNA extraction. The specific protocol can be downloaded from https://www.qiagen.com/cn/shop/sample-technologies/rna/exorneasy-serum-plasma-kits/#orderinginformation.

### Side population analysis

The cell suspensions were labeled with Hoechst 33342 (Molecular probes – #H-3570) dye for side population analysis as per standard protocol [61]. Briefly, cells were resuspended at 1× pre-warmed OptiMEM (Gibco, USA) containing 2% FBS (Gibco, USA) at a density of 10^6^/mL. Hoechst 33342 dye was added at a final concentration of 5 μg/mL in the presence or absence of verapamil (50 μmol/L; Sigma) and the cells were incubated at 37°C for 90 min with intermittent shaking. At the end of the incubation, the cells were washed with OptiMem containing 2% FBS and centrifuged down at 4°C, and resuspended in ice-cold OptiMem containing 2% FBS and 10 mmol/L HEPES. Propidium iodide (Sigma, USA) at a final concentration of 2l g/mL was added to the cells to gate viable cells. The cells were filtered through a 40 μm cell strainer to obtain single cell suspension before sorting. Analysis and sorting was done on a FACS AriaI (Becton Dickinson). The Hoechst 33342 dye was excited at 355 nm and its dual-wavelength emission at blue and red region was plotted to get the SP scatter.

### Spheroid formation assay

Cells (500 cells/well) were seeded into 6-well Ultra Low Cluster plates (Corning) and cultured in suspension in serum-free DMEM-F12 (BioWhittaker), supplemented with B27 (1:50, Invitrogen), 20 ng/mL endothelial growth factor (EGF; BD Biosciences), 0.4% bovine serum albumin (Sigma), and 4 mg/mL insulin (Sigma). After 10–12 days, the number of cell spheroids (tight, spherical, non-adherent masses > 50 μm in diameter) were counted, and images of the spheroids were scored under an inverse microscope (spheroids formation efficiency=colonies/input cells × 100 %).

### Flow cytometric analysis

Flow cytometric analyzed of apoptosis were used the FITC Annexin V Apoptosis Detection Kit I (BD, USA), and was presented as protocol described. Briefly, cells were dissociated with trypsin and resuspended at 1 × 10^6^ cells/mL in binding buffer with 50 μg/mL FITC Annexin V and 50 μg/mL PI. The cells were subsequently incubated for 15 minutes at room temperature, and then were analyzed by Gallios flow cytometer (Beckman Coulter, USA). The cell's inner mitochondrial membrane potential (Δψm) was detected by flow cytometric using MitoScreen JC-1 staining kit (BD), and was presented as protocol described. Briefly, cells were dissociated with trypsin and resuspended at 1 × 10^6^ cells/mL in Assay Buffer, and then incubated at 37°C for 15 minutes with 10 μg/mL JC-1. Before analyzed by flow cytometer, cells were washed twice by Assay Buffer. Flow cytometry data were analyzed using FlowJo 7.6 software (TreeStar Inc., USA).

### Caspase-9 or -3 activity assays

Activity of caspase-9 or -3 was analyzed by spectrophotometry using Caspase-9 Colorimetric Assay Kit (Cat#:C1157, Beyotime, China; website: http://beyotime.com/product/C1157.htm) or Caspase-3 Colorimetric Assay Kit (Cat#:C1115, Beyotime, China; website: http://beyotime.com/product/C1115.htm), and was performed as protocol described. Briefly, 5 × 10^6^ cells or 100 mg fresh tumor tissues were washed with cold PBS and resuspended in Lysis Buffer and incubated on ice for 30 min. Mixed the 50 μL cell suspension, 50 μL Reaction Buffer, and 5 μL Caspase-3/-9 substrate, and then incubated at 37°C for 4 hours. The absorbance was measured at 405 nm, and BCA protein quantitative analysis was used as the reference to normal each experiment groups.

### Tumor xenografts

All experimental procedures were approved by the Institutional Animal Care and Use Committee of Sun Yat-sen University. The 6-week-old BALB/c-nu mice were randomly divided into four groups (*n* = 6 per group) and the indicated cells (2 × 10^6^) were inoculated subcutaneously into the inguinal folds of the nude mice. After ten days for cells inoculation, the mice were injected with 150 μg agomir negative control, agomir-196b-5p, antagomir negative control and antagomir-196b-5p (2 mg/mL) three times each week for four weeks respectively, combined with intraperitoneal injection of 5-FU (50 mg/kg.d) for 4 weeks [62]. The dose and length of treatment by agomir negative control, agomir-196b-5p, antagomir negative control and antagomir-196b-5p were performed according to the manufacture's protocol (http://www.sirna.cn/sitecn/Product.aspx?id=75). Tumor volume was determined using an external caliper and calculated using the equation (L × W^2^)/2. On day 38, tumors were detected by an IVIS imagining system (Caliper, USA), then animals were euthanized, tumors were excised, weighed and stored in liquid nitrogen tanks.

### Luciferase assay

Cells (4 × 10^4^) were seeded in triplicate in 24-well plates and cultured for 24 h. Cells were transfected with 100 ng pSTAT3 reporter luciferase plasmid, or pmirGLO-SOCS1-3′UTR, or –SOCS3-3′UTR luciferase plasmid, plus 5 ng pRL-TK Renilla plasmid (Promega) using Lipofectamine 3000 (Invitrogen) according to the manufacturer's recommendation. Luciferase and Renilla signals were measured 36 h after transfection using a Dual Luciferase Reporter Assay Kit (Promega) according to the manufacturer's protocol.

### miRNA immunoprecipitation

Cells were co-transfected with HA-Ago2, followed by HA-Ago2 immunoprecipitation using HA-antibody. Real-time PCR analysis of the IP material was used to test the association of the mRNA of SOCS1 and SOCS3 with the RISC complex.

### Statistical analysis

All values are presented as means ± standard deviation (SD). Significant differences were determined using GraphPad 5.0 software (USA). Student's *t-test* was used to determine statistical differences between two groups. One-way ANOVA was used to determine statistical differences between multiple testing. The chi-square test was used to analyze the relationship between miR-196b-5p expression and clinicopathological characteristics. Survival curves were plotted using the Kaplan Meier method and compared by log-rank test. *P <* 0.05 was considered significant. All the experiments were repeated three times.

## SUPPLEMENTARY MATERIALS FIGURES AND TABLES



## Abbreviations

CRC: CRC
CSCs: cancer stem cells
STAT3: signal transducer and activator of transcription 3
SOCS: suppressor of cytokine signaling
TCGA: The Cancer Genome Atlas
IHC: immunohistochemistry
3′UTRs: 3′ untranslated region
SP: side population
NANOG: Nanog homeobox
BMI1: proto-oncogene polycomb ring finger
OCT4: OU class 5 homeobox 1A
5-FU: 5-fluorouracil
RIP assay: miRNA immunoprecipitation
