# Supplementary material for: Maintenance of cancer stemness by miR-196b-5p contributes to chemoresistance of colorectal cancer cells via activating STAT3 signaling pathway
Source: Oncotarget. 2017 May 18;8(30):49807–23. doi: 10.18632/oncotarget.17971 (PMC5564809; doi:10.18632/oncotarget.17971)
Supplement: Supplementary file 1 [file oncotarget-08-49807-s001.pdf]

Maintenance of cancer stemness by miR-196b-5p contributes to chemoresistance of colorectal cancer cells via activating STAT3 signaling pathway

Supplementary Materials

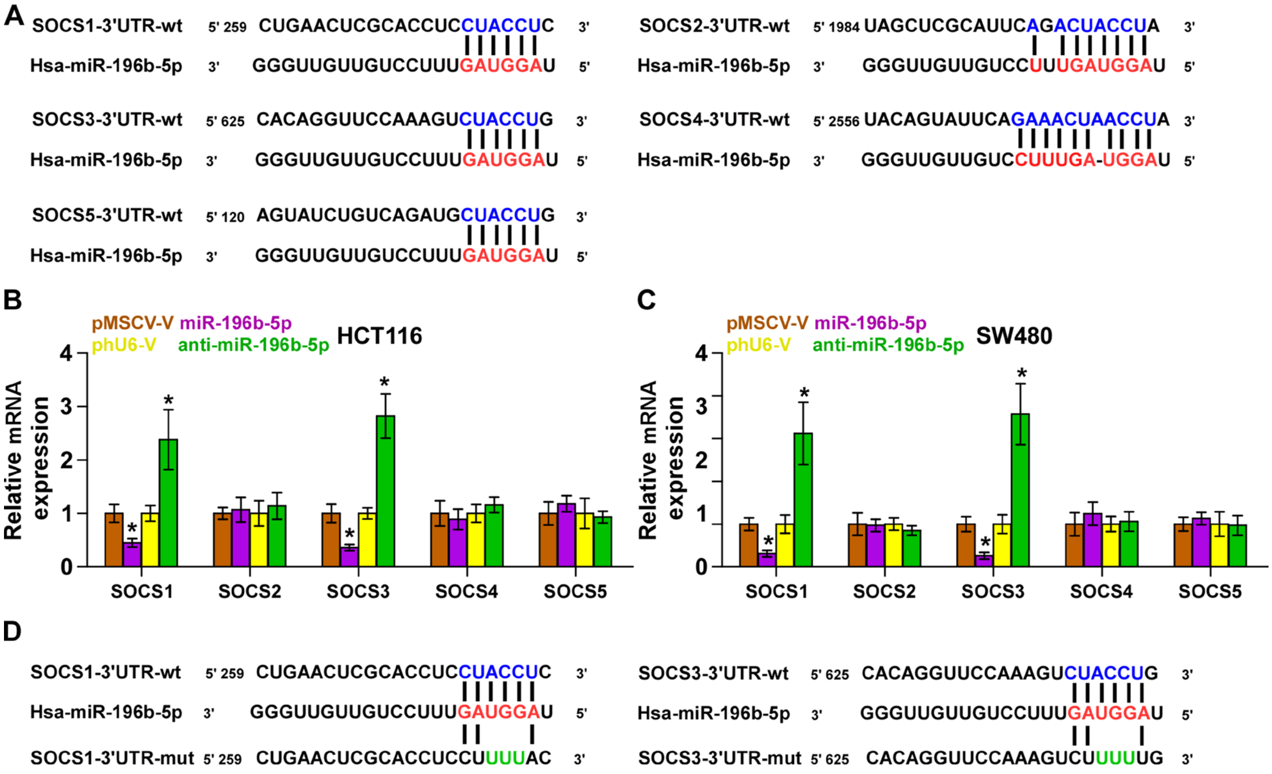

Supplementary Figure 1: (**A**) Predicted miR-196b-5p targeting sequence in 3′UTRs of SOCS1-5. (**B** and **C**) Real-time PCR analysis of SOCS1-5 expression in the indicated HCT116 and SW480 cells. Transcript levels were normalized by GAPDH expression. Error bars represent the mean ± s.d. of three independent experiments. *$P < 0.05$. (**D**) Mutant sequence of 3′UTRs of SOCS1 and SOCS3.

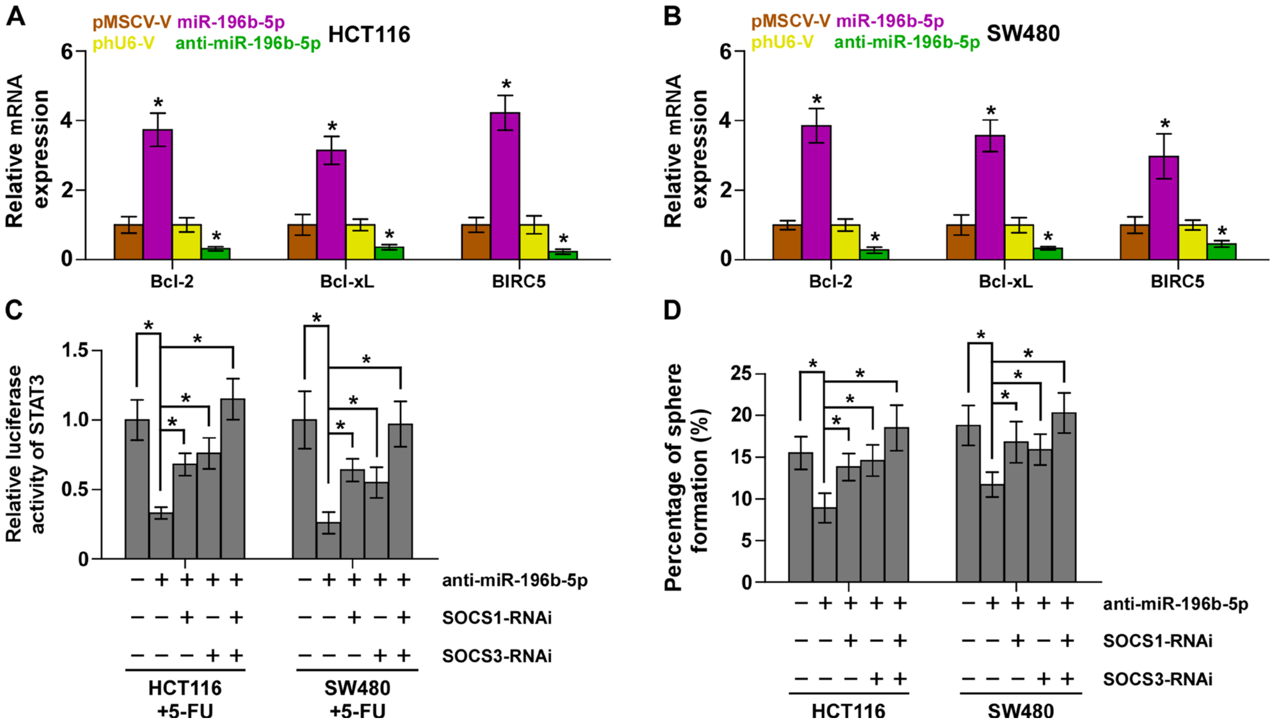

Supplementary Figure 2: (**A** and **B**) Real-time PCR analysis of Bcl2, Bcl-xL and BIRC5 in the indicated cells. Transcript levels were normalized by GAPDH expression. Error bars represent the mean ± s.d. of three independent experiments. *$P < 0.05$. (**C**) Individual silencing of SOCS1 and SOCS3, or simultaneously silencing SOCS1 and SOCS3 rescued the STAT3 activity repression in miR-196b-5p-silencing cells. Error bars represent the mean ± s.d. of three independent experiments. *$P < 0.05$. (**D**) Individual silencing of SOCS1 and SOCS3, or simultaneously silencing SOCS1 and SOCS3 rescued the sphere formation repressed by anti-miR-196b-5p. Error bars represent the mean ± s.d. of three independent experiments. *$P < 0.05$.

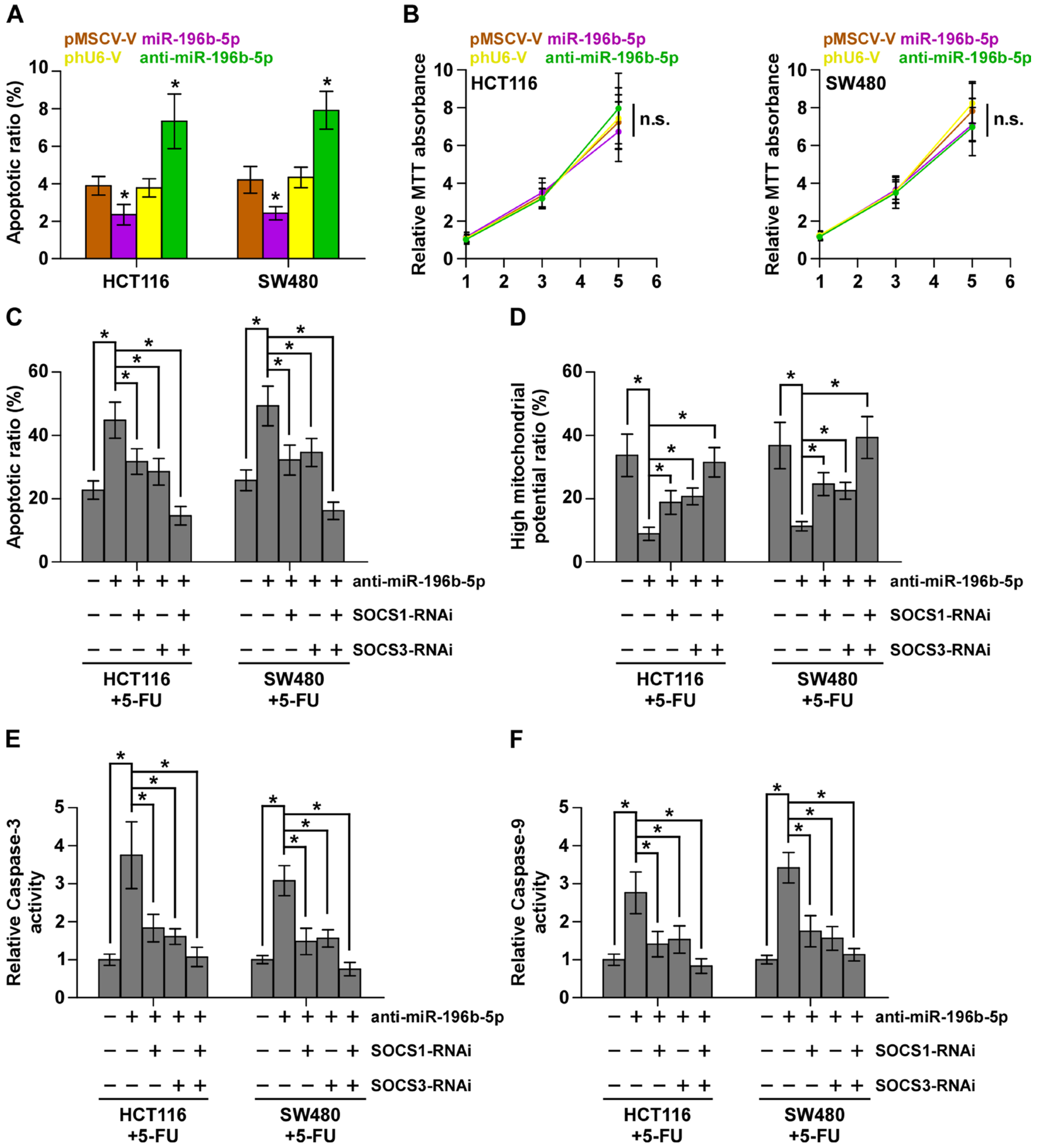

Supplementary Figure 3: (**A**) Upregulating miR-196b-5p decreased, whilie silencing miR-196b-5p increased the apoptotic ratio in the absence of 5-FU. Error bars represent the mean ± s.d. of three independent experiments. **P* < 0.05. (**B**) The effect of miR-196b-5p on proliferation of CRC cells was assessed by MTT assay. Error bars represent the mean ± s.d. of three independent experiments. (**C**) Individual silencing of SOCS1 and SOCS3, or simultaneously silencing SOCS1 and SOCS3 rescued the apoptotic rateof colorectal cancer cells increased by anti-miR-196b-5p. Error bars represent the mean ± s.d. of three independent experiments. **P* < 0.05. (**D**) Individual silencing of SOCS1 and SOCS3, or simultaneously silencing SOCS1 and SOCS3 rescued the mitochondrial potential of colorectal cancer cells repressed by anti-miR-196b-5p. Error bars represent the mean ± s.d. of three independent experiments. **P* < 0.05. (**E** and **F**) Individual silencing of SOCS1 and SOCS3, or simultaneously silencing SOCS1 and SOCS3 rescued the caspase-3 or -9activity of colorectal cancer cells increased by anti-miR-196b-5p. Error bars represent the mean ± s.d. of three independent experiments. **P* < 0.05.

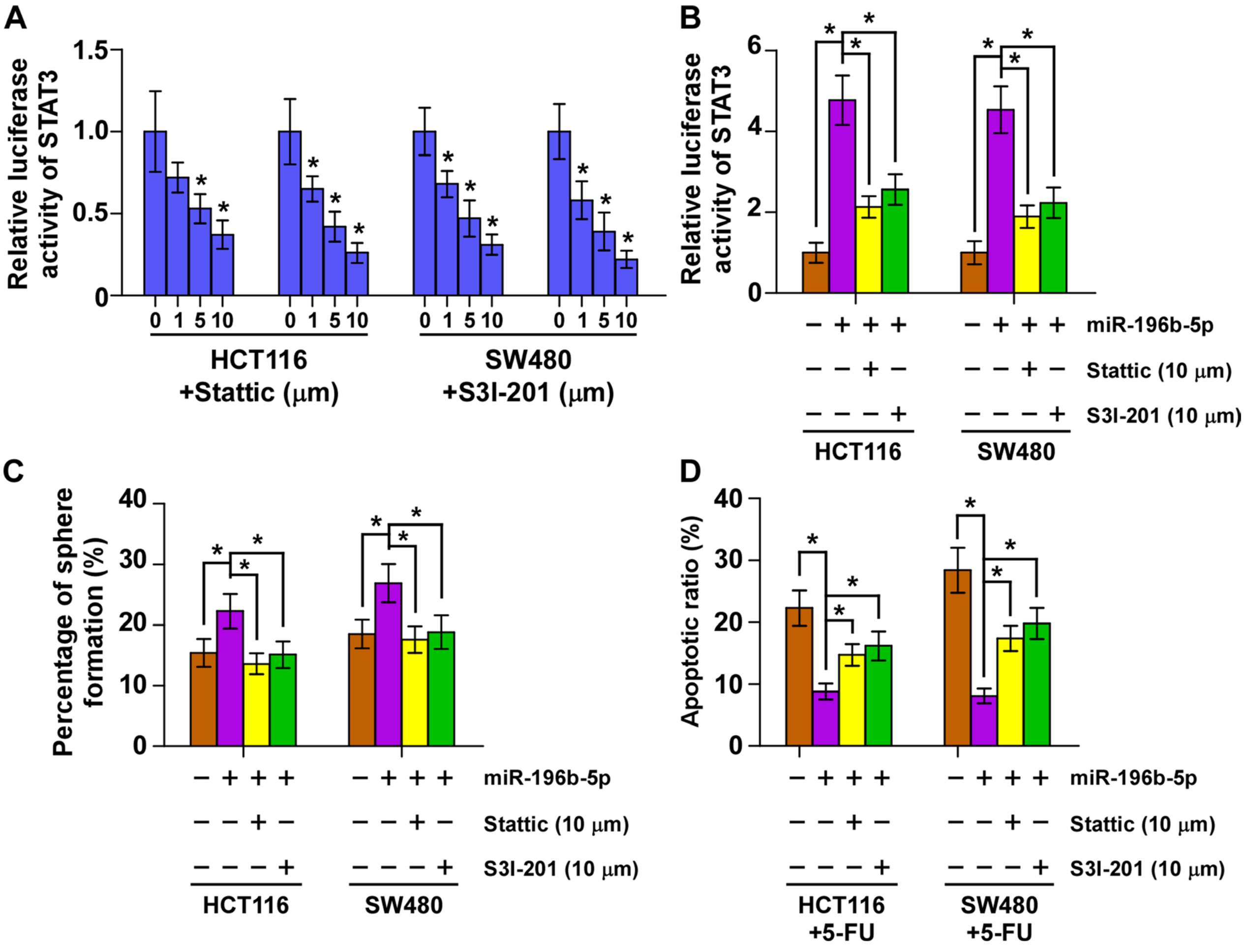

Supplementary Figure 4: miR-196b-5p promotes stemness and chemoresistance via activating STAT3 signaling. (A) The inhibitors of STAT3 signaling Stattic and S3I-201 repress STAT3 activity in a dose-dependent manner in the indicated CRC cells. Error bars represent the mean ± s.d. of three independent experiments. *$P < 0.05$. (**B**) STAT3 activity under treatment of the inhibitors Stattic (10 μM) and S3I-201 (10 μM) in the indicated cells. Error bars represent the mean ± S.D. of three independent experiments. *$P < 0.05$. (**C**) Spheroid formation abilities of the indicated cells after treated with Stattic and S3I-201 respectively. Error bars represent the mean ± s.d. of three independent experiments. *$P < 0.05$. (**D**) Annexin V-FITC/PI staining of the indicated cells after treated with Stattic and S3I-201 respectively. Error bars represent the mean ± s.d. of three independent experiments. *$P < 0.05$.

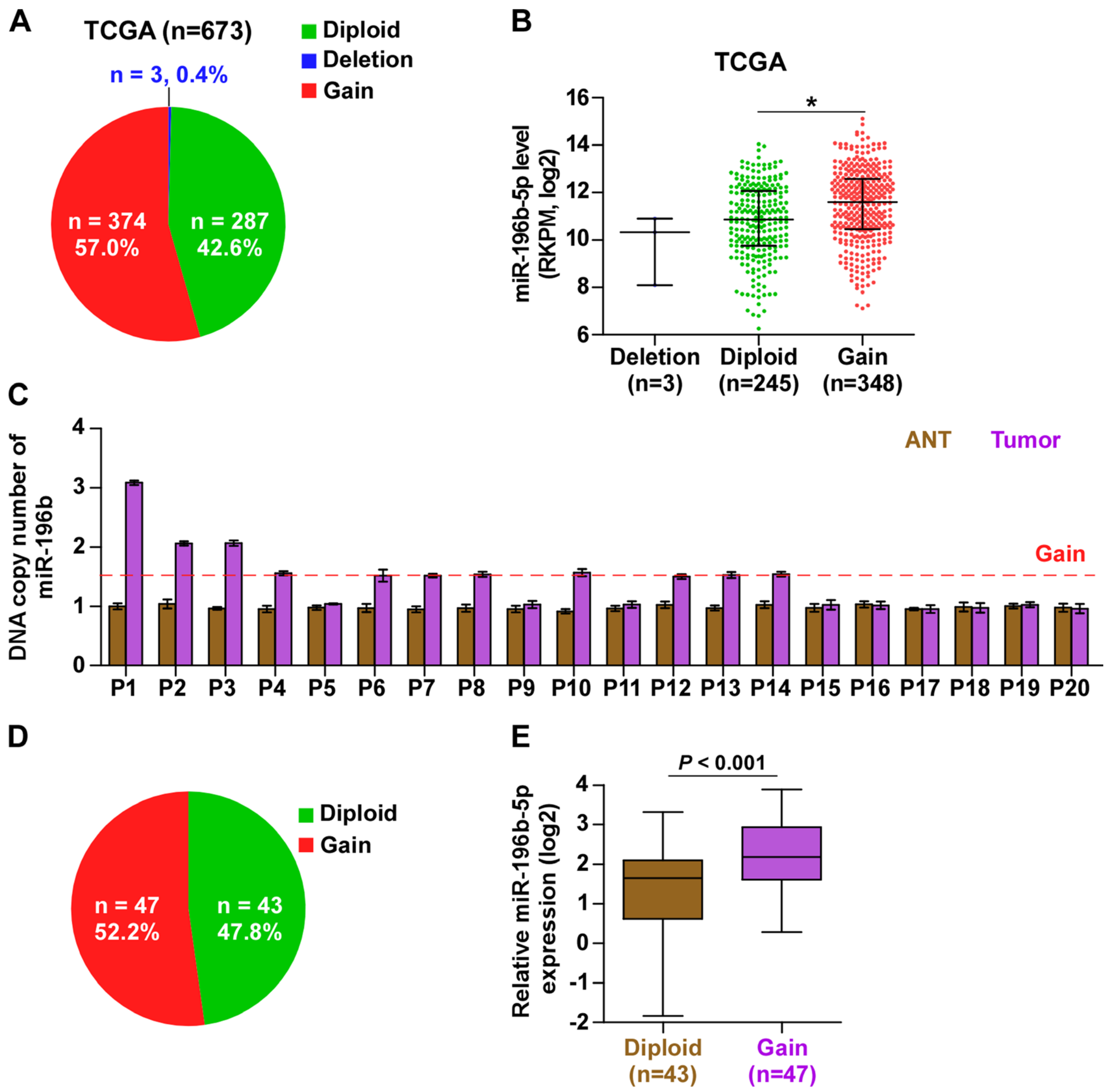

Supplementary Figure 5: Recurrent gains are involved in miR-196b-5p overexpression in CRC tissues. (**A**) The percentage of miR-196b-5p with gains in the CRC tissues from TCGA. (**B**) The expression level of miR-196b-5p in CRC tissues with gains was higher than those without gains through analyzing TCGA CRC datasets. *$P < 0.05$. (**C**) The copy-number variation of miR-196b-5p in 20 paired CRC patients. Each bar represents the mean values ± SE. (**D**) The percentage of miR-196b-5p with gains in our clinical CRC tissues. (**E**) The expression level of miR-196b-5p in CRC tissues with gains was higher than those without gains in our CRC tissues. Each bar represents the median values ± quartile values. $P < 0.001$.

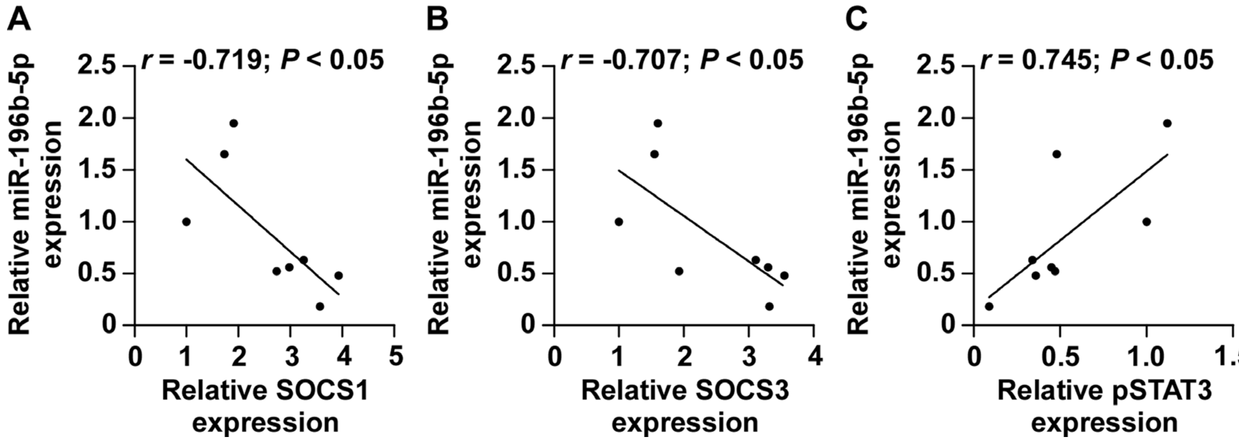

Supplementary Figure 6: Clinical correaltion of miR-196b-5p with SOCS1, SOCS3 and nuclear pSTAT3 in humanCRC tissues. (**A–C**) Correlation between miR-196b-5p expression levels and SOCS1, SOCS3 and nuclear pSTAT3 expression in CRC tissues. The expression levels of SOCS1, SOCS3 and nuclear pSTAT3 were quantified by densitometry using Quantity One Software, and normalized to the levels of α-tubulin and p84, respectively. The sample 1 was used as a standard. The relative expressions of miR-196b-5p and these proteins were used to perform the correlation analysis.

Supplementary Table 1: The clinicopathological characteristics in patients with colorectal cancer

Parameters	Number of cases	
	Group 1 (n = 90)	Group 2 (n = 150)
Gender		
Female	37	49
Male	53	101
Age (years)		
< 65	44	71
≥ 65	46	79
Location		
Colon	64	94
Rectum	26	56
Survival status		
Alive	55	143
Dead	35	7
Clinical stage		
I–II	53	115
III–IV	37	35
T stage		
$T_1 - T_2$	21	45
$T_3 - T_4$	69	105
N stage		
N_0	57	108
N_{1-2}	33	42
M stage		
M_0	74	121
M_1	16	29

Supplementary Table 2: The relationship between miR-196b-5p expression in tissues and clinicopathological characteristics in 90 patients with colorectal cancer

Parameters	Number of cases	miR-196b-5p expression		*P* values
		Low	High	
Gender				
Female	37	20	17	0.521
Male	53	25	28	
Age (years)				
< 65	44	20	24	0.399
≥ 65	46	25	21	
Location				
Colon	64	31	33	0.642
Rectum	26	14	12	
Clinical stage				
I–II	53	28	25	0.521
III–IV	37	17	20	
T stage				
T_1–T_2	21	14	7	0.081
T_3–T_4	69	31	38	
N stage				
N_0	57	29	28	0.827
$N_{1–2}$	33	16	17	
M stage				
M_0	74	41	33	0.027*
M_1	16	4	12	

Supplementary Table 3: The clinicopathological characteristics in 90 healthy volunteers without cancer or infectious diseases

Parameters	Number of cases	Parameters	Number of cases
Gender		Age (years)	
Female	32	< 65	25
Male	58	≥ 65	35

Supplementary Table 4: The relationship between miR-196b-5p expression in the serum exosomes and clinicopathological characteristics in 150 patients with colorectal cancer

Parameters	Number of cases	miR-196b-5p expression		*P* values
		Low	High	
Gender				
Female	49	26	23	0.273
Male	101	49	52	
Age (years)				
< 65	71	31	40	0.141
≥ 65	79	44	35	
Location				
Colon	94	44	50	0.311
Rectum	56	31	25	
Clinical stage				
I–II	115	61	54	0.176
III–IV	35	14	21	
T stage				
$T_1 – T_2$	45	16	29	0.021*
$T_3 – T_4$	105	59	46	
N stage				
N_0	108	50	58	0.146
$N_{1–2}$	42	25	17	
M stage				
M_0	121	71	50	< 0.001*
M_1	29	4	25	

Supplementary Table 5: A list of primers used in the reactions for real-time RT-PCR

**Real-time PCR primer:**	
SOCS1-up	CACATGGTTCCAGGCAAGTA
SOCS1-dn	CTACCTGAGCTCCTTCCCCT
SOCS2-up	GGAGGACGGATGACAAAGTC
SOCS2-dn	AGACACTCTCCGGACTGAGG
SOCS3-up	CAAGGACGGAGACTTCGATT
SOCS3-dn	AACTTGCTGTGGGTGACCAT
SOCS4-up	TTCTTCTGGGCACTTTCTGG
SOCS4-dn	AGACTGATGGCGATGGTGAT
SOCS5-up	CTCCCTCATGACCGAAGAGA
SOCS5-dn	TAAAGCCCTGCCTGCTATTG
BCL2L1-up	GGTATTGGTGAGTCGGATCG
BCL2L1-dn	TGCTGCATTGTTCCCATAGA
BCL2-up	GTGGATGACTGAGTACCTGAACC
BCL2-dn	AGACAGCCAGGAGAAATCAAAC
BIRC5-up	CCATTAACCGCCAGATTTGA
BIRC5-dn	TGTAGAGATGCGGTGGTCCT
GAPDH-up	ATTCCACCCATGGCAAATTC
GAPDH-dn	TGGGATTTCCATTGATGACAAG

Supplementary Table 6: A list of primers used in the reactions for clone PCR

**Used for subcloning and plasmid construction:**	
miR-196b-clone-F	ATCCTTCCTAGTCCAGCCTGAG
miR-196b-clone-R	ACCTGGCGGCACTCCTTA
SOCS1-3UTR-17nt-clone-F	TGCACGCAGCATTAACTGG
SOCS1-3UTR-373nt-clone-R	TAAAATAGGATTCTGCACAGCAGA
SOCS3-3UTR-44nt-clone-F	GCAAAGTTTGACTTGGATTGG
SOCS3-3UTR-1585nt-clone-R	CGTGGCACATGGCACAA
